# Motivation in motor training interventions after stroke—a systematic review

**DOI:** 10.3389/fresc.2026.1754746

**Published:** 2026-05-28

**Authors:** M. Schrader, M. Wiest, A. Sterr, C. Dohle

**Affiliations:** 1 Research Department, Fürst Donnersmarck Foundation of Berlin, Berlin, Germany; 2 Center for Stroke Research Berlin, Charité – Universitätsmedizin Berlin, Berlin, Germany; 3 P.A.N. Center for Post-Acute Neurorehabilitation, Berlin, Germany

**Keywords:** motivation, motor performance, neurorehabilitation, stroke, systematic review

## Abstract

**Objective:**

Motivation plays an important role in the rehabilitation process and can significantly influence its success. The objective of this systematic review was to identify which therapy interventions targeting motor improvement after stroke have also been evaluated in terms of their motivational effects.

**Method:**

A comprehensive literature search was conducted in August 2025 using the databases CINAHL, MEDLINE, Embase and PEDro. Randomised controlled trials were eligible for inclusion if they reported at least one outcome related to both motor performance and motivation. Relevant trials were identified, screened, and summarized in accordance with the PRISMA reporting guidelines. For each assessment, the direction of effect was estimated. The review was prospectively registered in PROSPERO (Trial-No. CRD42023448438).

**Results:**

Overall, motivation was measured only rarely. 17 trials including a total of 521 stroke survivors met the inclusion criteria. In general, all included studies showed a high risk of bias due to the inability to blind the intervention. Exergaming was the most frequently investigated intervention, others described animal-assisted therapy, smart technology and boxing. Four exergame trials and one animal-assisted trial demonstrated beneficial effects of the intervention on motivation and motor performance.

**Conclusion:**

Current evidence on the impact of motor interventions after stroke on motivation is weak. Exergames seems to have promising potential to motivate stroke patients and improve motor performance; however, the underlying mechanisms remain insufficiently understood. Social interaction, effective in one animal-assisted therapy, represents another promising avenue. Overall, motivation and its standardized assessment warrant greater research attention to enhance therapeutic outcomes.

## Introduction

Stroke is one of the leading cause of permanent disability in adulthood (1). Among the various post-stroke symptoms, motor impairments such as loss of functional movement are particularly common (2), and executing deliberate and controlled movements becomes challenging (3). Within the framework of the International Classification of Functioning, Disability and Health (ICF) (4), motor impairments are predominantly classified as neuromusculoskeletal and movement-related functions within the domain of ‘Body function and Structures’. This includes, for example, coordination of movement or muscle strength. In addition, ICF distinguishes motor performance at the level of activities, with can be found in the category of mobility within the ICF domain ‘Activities and Participation’, comprising walking, changing body positions, transfers, as well as grasping and holding objects. In the context of this study, the term motor performance is used, encompassing components of both motor impairment and motor function.

To restore functional movement, task-oriented, high-frequency exercise is essential (5–8). Several evidence-based approaches exist for improving motor performance, mainly based on intensive training (9). However, such intensive and highly repetitive training can be fatiguing and demands a high degree of motivation to maintain active engagement in rehabilitation training. Motivation can be defined as ‘the process whereby goal-directed activities are instigated and sustained’ (10). In the context of this review, motivation refers to patients' engagement and participation in motor rehabilitation training rather than to psychological or psychotherapeutic interventions aimed at modifying motivation. Patients with acquired brain injury often exhibit impairments in goal-directed, motivated behaviour, which poses challenges both for the individual and the rehabilitation process (11). In the domain of the upper extremity, stroke patients with more severe motor impairments have been reported to show lower rehabilitation motivation compared to those with milder impairments (12). Therefore, motivation plays a crucial role in rehabilitation, and enhancing motivation can influence the success of rehabilitation efforts (12–14).

From a psychological perspective, motivation can be driven either intrinsically or extrinsically, which differ in terms of the degree of self-determination (15). Intrinsic motivation stems from internal drivers such as enjoyment, curiosity or a pursuit of a new challenge, whereas extrinsic motivation arises from external incentives, such as rewards (15). The concept of motivation encompasses various constructs (16) such as engagement, fun and enjoyment. In the context of rehabilitation, it is assumed that therapies need to incorporate these characteristics in order to maintain a patient's motivation to actively participate in therapeutic programs (17). Closely related concepts such as adherence and self-efficacy are sometimes used interchangeably with motivation. In this context, adherence refers to whether, and to what extent, a person maintains a behaviour recommended by a health care provider (18), which can be considered a marker of motivation (13, 19). Self-efficacy, on the other hand, describes an individual's belief in their ability to achieve specific outcomes (20). In sum, motivation is conceptualized in many terms, and consequently, can be measured in different ways. Although reliable and valid instruments exist for measuring the different constructs and types of motivation (21, 22), they appear to be underutilized in clinical trials (17, 23).

In stroke rehabilitation, various motor rehabilitation approaches—such as exergames (a combination of the words ‘exercise’ and ‘games’), virtual reality or robotic are frequently promoted with claims of enhancing patient motivation. However, these assertions of motivational benefits often resemble marketing rhetoric rather than being consistently substantiated by robust empirical evidence, leaving their validity uncertain. Currently, motor performance rehabilitation lacks a comprehensive overview of therapies that both enhance motor function or motor impairment and effectively motivate stroke patients. The systematic review aims to address this gap. The objective was to identify therapy interventions that measure motor performance and motivation after stroke, as well as to evaluate their effects in regard to improving motor performance and motivation.

## Method

### Protocol and registration

This systematic review was reported according to the Preferred Reporting Items for Systematic Reviews and Meta-Analyses (PRISMA) guidelines and its associated checklist (24). The protocol was registered on PROSPERO after the development of the search strategy and before data extraction and analysis (https://www.crd.york.ac.uk/prospero/Trial No. CRD42023448438). The systematic review does not have a requirement for ethics approval.

### Outcomes

We considered the following assessments of motivation and motor performance in this review.

For motivation, the Intrinsic Motivation Inventory (IMI) (25), the Self-Regulation of Motivation Scale (SRMS) (26), the Physical Activity Enjoyment Scale (PACES) (27), adherence measures, the Visual Analog Scale (VAS), as well as motivation-based items from standardized or self-developed questionnaires were included.

For motor performance we included assessments of motor function as well as motor impairment. We included tests that assess motor impairments of body functions, which are assigned to the ICF component ‘Body Functions and Structures’, especially Fugl-Meyer-Test (28), Range of Motion (ROM), Trunk Impairment Scale (29), Hand strength. Motor function, describes the ability to actually perform motor tasks and activities and belongs to the ICF component ‘Body Function’ as well as ‘Activities and Participation’. Tests for this domain included Action Research Arm Test (ARAT) (30), Box & Block Test (31), Instrumental Activities of Daily Living (IADL) (32), Stroke Impact Scale (SIS) (33), Timed up and Go Test (TUG) (34), Wolf Motor Function Test (WMFT) (35), Nine Hole Peg Test (NHPT) (36).

Further assessments addressing motivation and motor performance were included if deemed relevant.

### Inclusion and exclusion criteria

This systematic review focuses on training-based motor interventions directed towards the upper or lower extremities, or trunk control. Trials were included if they (1) were randomised controlled trials (RCT) or cross-over trials (CT); (2) measured both motivation and motor performance as outcomes; in this context, motivation was operationalized through outcomes assessing constructs such as engagement, enjoyment, adherence, or self-efficacy related to rehabilitation training; whereas motor performance was operationalized through assessments of motor impairment or motor function; (3) examined adult patients with stroke (aged >18 years); (4) published in any language in peer-reviewed journals. Trials were excluded if (1) results on stroke patients were not reported separately; (2) patients participated in the early acute phase (<2 weeks after onset); (3) the interventions were carried out only once; (4) motivation was measured only in one (intervention or control) group; (5) they used neuromodulation (e.g., repetitive transcranial magnetic stimulation) or educational (e.g., behavioural training) interventions; (6) they were review trials, trial protocols, conference papers or ongoing trials.

### Search strategy

A literature search was performed in August, 2025, using the following databases: CINAHL via EBSCO, MEDLINE via Ovid, Embase via Ovid and PEDro. The reference lists of included and further relevant articles were manually searched to identify additional trials.

Two search terms were central to the review: (a) For *motivation* we included the terms of engagement, enjoyment, fun, adherence and self-efficacy. (b) *Motor function* was based on terms commonly used in neurorehabilitation (7, 37, 38). Specifically, the search terms were: motivation OR engagement OR patient adherence OR enjoyment OR self-efficacy OR fun OR mood AND stroke OR cerebrovascular disorder AND motor function OR hemiparesis OR functional abilities OR upper extremity OR arm OR lower extremity OR leg OR gait OR walking OR ambulation OR locomotion OR mobility OR balance OR standing OR cycling OR transfer OR falls OR range of motion AND therapy OR treatment OR training OR physical therapy modalities OR occupational therapy OR physiotherapy OR rehabilitation. The Cochrane Embase randomized controlled trial filter was used (39). The entire search strategy is presented in the Supplementary Table S1.

### Study selection

**Figure 1:**
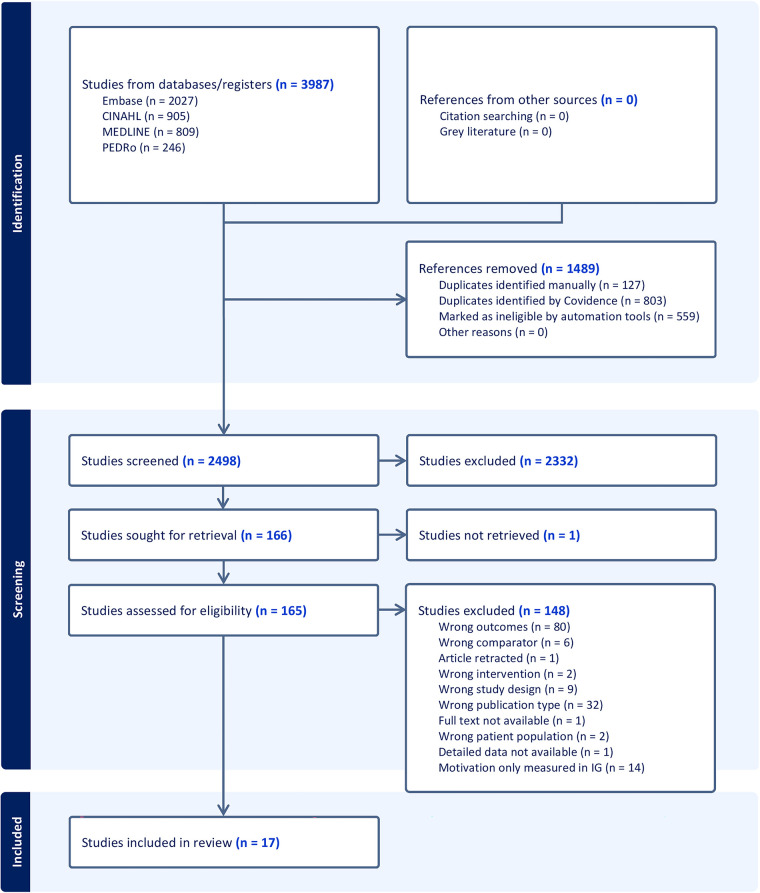
PRISMA chart.

The screening process is illustrated in Figure 1. After executing the database searches, all identified records were imported into Covidence software (40) for subsequent processing. Initially, duplicates were removed. Two independent reviewers (MS and MW) then screened titles and abstracts for relevance, excluding irrelevant trials. Full texts of potentially eligible articles were independently assessed for inclusion by the same two reviewers (MS and MW). Discrepancies were resolved through discussion until consensus was reached; if disagreements persisted, a third reviewer (AS) was consulted at any stage of the screening process.

### Risk of bias assessment

Risk of bias of the included trials was evaluated using the Rob2 tool (41), which assesses five domains: (1) bias arising from the randomization process; (2) bias due to deviations from intended interventions; (3) bias due to missing outcome data; (4) bias in outcome measurement; and (5) bias in the selection of the reported result. The reviewers (MS and MW) independently rated each trial. Trials were classified as ‘high risk of bias’ if at least one domain was rated as ‘high risk’ or if multiple domains raised ‘some concerns’ (41).

### Data extraction and data synthesis

Aggregate data from included trials were extracted using a predefined charting framework developed by the authors. This framework was pilot-tested on a sample of three trials to verify inter-rater agreement. Data extraction was conducted independently by two reviewers (MS, MW). Corresponding authors were contacted to obtain missing data. Discrepancies in data extraction were resolved via discussion or by involving a third reviewer (AS). Extracted information comprised trial and participant characteristics, intervention and control conditions. To capture the diversity of the assessment landscape, the primary endpoints and all other assessment tools employed by the respective authors were extracted and systematically noted. Numerical values for age and time since stroke were recorded as means (m) and standard deviations (SD). Where median (md) and interquartile range (IQR) for age and time since stroke were reported, means and standard deviation were estimated following the method of Hozo et al. (42). For cross-over trials, only data from the first intervention phase were included in the analysis (43). In three-arm trials, each pairwise comparison was analysed separately. When self-developed questionnaires were used, only motivation-related items were considered. This systematic review focused on the immediate pre-post time frame; for trials reporting follow-ups, only the pre-post data were considered.

Due to the heterogeneity of the data, particularly regarding motivation-related assessments and the joint evaluation of two outcomes, conduction of a meta-analysis was not feasible. Instead, the method of vote counting based on the direction of effect (‘favour intervention’, ‘no effect’, ‘favour control’) was applied to evaluate effects (44–46). The judgement was based on the statistical significance of the difference between the groups in the respective domains. For assessments with multiple items or subscales, a majority rule was applied; for example, if the majority of items, e.g., three out of five indicated a significant intervention effect, the overall rating was ‘favour intervention’, whereas a single significant item out of five led to a rating of ‘no effect’. If there is a tie, the decision will be made in favour of the intervention.

## Results

### Characteristics of the included trials

The database search yielded 3987 records, of which 2498 remained after duplicate removal for title and abstract screening (Figure 1). Following title and abstract screening, 2332 articles were excluded as irrelevant, resulting in 166 full-texts for detailed assessment. 80 trials were excluded because they did not assess motivation as an outcome. Ultimately, 17 trials met all inclusion criteria and were included into the review (47–63). Trial characteristics are summarized in Table 1.

**Table 1:** Trial characteristics.

Trial	N (M/F)	Age years, m (SD)	Time since stroke, m (SD)	Intervention/Control	Dose/duration	Effect direction motivation	Effect direction motor function	Effect direction motor impairment
Ahmad 2019	36 (31/5)	*I*: 57.0 (8.2) *C*: 62.9 (10.5)	*I*: 10.6 (5.3) *C*: 10.7 (6.0) months	VR game + PT/ Conventional PT	*I*: 0,5 h + 1,5 h *C*: 2 h / 1 per week, 8 weeks	IMI ◄►	IADL ◄► SIS Hand ◄► SIS Mobility ◄► WMFT ◄►	Fugl-Meyer ◄► SIS Strenght ◄►
An 2021	30 (18/12)	*I*: 60.9 (8.2) *C*: 63.9 (7.7)	*I*: 12.9 (3.5) *C*: 13.6 (3.6) months	Walking with dogs/ Conventional walking	*I*: 30 min *C*: 30 min/ 1 per week, 8 weeks	SRMS ▴	Cadence ▴ Gait Speed ▴	Symmetric Index ▴ Stride Length ▴
Bergmann 2018	20 (14/6)	*I*: 62.0 (11.0) *C*: 65.0 (8.0)	*I*: 11.0 (5.0) *C*: 11.0 (3.0) months	VR augment robot-assisted gait training/ Robot-assisted gait training	*I*: 20–60 min *C*: 20–60 min/ 3 per week, 4 weeks	Adherence ◄► IMI ▴ ^3^	FAC ◄►	
Emmerson 2017	62 (39/23)	*I*: 68.0 (15.0) *C*: 63.0 (18.0)	*I*: 122 (77–193) *C*: 133 (58–228)^1^ days	Video home exercise with smart technology/ Paper-based home exercise	not specified/ 1–2 daily, 4 weeks	Adherence ◄► Enjoyment ◄►	WMFT ◄►	
Fluet 2024	28 (22/6)	*I*: 58 (11.1) *C*: 55.87 (14.5)	*I*: 29.15 (28.8) *C*: 63 (84) months	Scaffolding game-based home program/Game-based home program	*I*: ≥ 20 min *C*: ≥ 20 min/ 7 per week, 10 weeks	Adherence ◄► IMI ◄►	ARAT ◄► SIS Hand ◄►	Fugl-Meyer ◄►
Folkerts 2017^1^	11 (9/2)	*I*: 58.2 *C*: 54.3	*I*: 23.4 *C*: 21.7 months	Game-based task-oriented strength training/ Eccentric strength training	*I*: 30 min *C*: 30–60 min/ 3 per week, 4 weeks	IMI ◄►	ARAT ◄►	Strength ◄►^2^
Friedmann 2014^1^	12 (7/5)	Overall: 57.0 (30.5)	Overall: 34.6 (32.5) months	a) Instrumented music glove/ Conventional hand tabletop exercises	*I*: 45 min *C*: 45 min/ 3 per week, 2 weeks	IMI ▴^3^ Enjoyment ▴ Motivation ▴	ARAT ◄► Box & Block ▴ NHPT ▴ WMFT ◄►	Fugl-Meyer ◄►
				b) Instrumented music glove/ Iso Trainer	*I*: 45 min *C*: 45 min/ 3 per week, 2 weeks	IMI ▴^3^ Enjoyment ▴ Motivation ▴	ARAT ◄► Box & Block ◄► NHPT ◄► WMFT ◄►	Fugl-Meyer ◄►
				c) Iso Trainer/ Conventional hand tabletop exercises	*I*: 45 min *C*: 45 min/ 3 per week, 2 weeks	IMI ◄►^3^ Enjoyment ◄► Motivation ◄►	ARAT ◄► Box & Block ◄► NHPT ◄► WMFT ◄►	Fugl-Meyer ◄►
Hung 2014	30 (18/10)	*I*: 55.4 (10.0) *C*: 53.4 (10.0)	*I*: 21.0 (11.3) *C*: 15.9 (8.0) months	Balance training with Wii fit/ Conventional weight shift training	*I*: 30 min *C*: 30 min/ 2 per week, 12 weeks	PACES ▴	TUG ◄►	Static Balance ◄► Forward Reach ◄►
Kerdsawat-mongkon 2023	18 (9/9)	*I*: 66.4 (6.5) *C*: 62.6 (7.0)	*I*: 24.0 (12.5, 42.0) *C*: 20.0 (6.5, 31.0)^2^ months	Boxing + balance and trunk exercises/ Balance and trunk exercises	*I*: 60 min *C*: 60 min/ 3 per week, 6 weeks	PACES ◄►^3^	Mini-Best Test ◄►	Trunk-Impairment-Scale ◄►
Kottink 2014	18 (13/5)	*I*: 65.3 (6.5) *C*: 58.4 (14.8)	*I*: 35.0 (20.2) *C*: 43.7 (28.7) months	Reach training game/ Conventional reach training	*I*: 30 min *C*: 30 min/ 3 per week, 6 weeks	IMI ◄►	ARAT ◄►	Fugl-Meyer ◄►
Kuo 2023	37 (28/9)	*I*: 57.5 (7.0) *C*: 59.5 (10.7)	<1 years (*I*: 6, *C*: 1); 1–3 years (*I*: 8, *C*: 8); 3–5 years (*I*: 3, *C*: 5); >5 years (*I*: 2, *C*: 4) years (n)	VR games/ Conventional OT	*I*: 60 min *C*: 60 min/ 2 per week, 9 weeks	PACES ▴	Box & Block ▴ IADL ◄► SIS Hand ◄►^3^	Active ROM ◄►^3^ Fugl-Meyer ◄► Strength ◄►
Nijenhuis 2017	20 (10/9)	*I*: 58 (48–65) *C*: 62 (54–70)^1^	*I*: 11.0 (10.0–26.0) *C*: 12.0 (10.0–30.0)^2^ months	Gaming home exercise with passive hand orthosis/ Conventional home exercise	*I*: 30 min *C*: 30 min/ 6 per week, 6 weeks	Adherence ▾ IMI ◄►	ARAT ◄► Box & Block ◄► Motor Act. Log ◄► SIS ◄►	Fugl-Meyer ◄► Strength ◄►
Park 2019	43 (26/17)	*I*: 56.9 (13.3) *C*: 62.0 (10.2)	*I*: 3.9 (1.6) *C*: 3.6 (1.1) months	Game-based hand resistance exercise/ Conventional OT	*I*: 30 min *C*: 30 min/ 5 per week, 6 weeks	Fun (VAS) ▴ Motivation (VAS) ▴	Box & Block ▴ M. Function Test ▴	Strength ▴
Popovic 2014	20 (n.a.)	*I*: 58.0 (8.0) *C*: 57.0 (12.0)	*I*: 17.0 (14.0) *C*: 20.0 (14.0) months	Feedback-mediated game-based drawing exercise/ Drawing exercise on a paper sheet	*I*: 25 min *C*: 25 min/ 5 per week, 5 weeks	Adherence ▴ IMI ◄►^3^	Modified-Drawning-Test ▴^3^	
Pouplin 2023	28 (15/13)	*I*: 55.8 (15.7) *C*: 67.8 (7.5)	*I*: 12.5 (11.8) *C*: 9.42 (4.7) months	Serious game training/ Home-based self-training via booklet	*I*: 30 min *C*: 30 min/ 5 per week, 2 weeks	Motivation ◄► (VAS)	Box & Block ◄► Frenchay ◄► WMFT ◄►	Fugl-Meyer ◄►
Prange 2015	68 (n.a.)	*I*: 60.3 (9.7) *C*: 58 (11.4)	*I*: 7.3 (3.4) *C*: 6.8 (3.1) weeks	Arm support training games/ Conventional reach training	*I*: 30 min *C*: 30 min/ 3 per week, 6 weeks	IMI ◄►^3^	Reach Distance ◄► SULCS ◄►	Fugl-Meyer ◄►
Segura 2024	40 (31/9)	*I*: 64.1 (10.5) *C*: 65 (12.1)	*I*: 2.8 (2.9) *C*: 1.8 (6.2) years	Enriched music-supported therapy/ Upper limb motor exercises	*I*: 60 min *C*: 60 min 4 per week, 10 weeks	IMI ◄►	ARAT ◄► Box & Block ◄► Chedoke ◄► NHPT ◄►	Fugl-Meyer ▴ Strength ◄►

M, men; F, female; m, mean; SD, standard deviation; PT, physio therapy; OT, occupational therapy; ARAT, Action Research Arm Test; Chedoke, Chedoke Arm and Hand Activity Inventory; FAC, Functional Ambulation Categories; Frenchay, Frenchay Activity Index; IADL, Instrumental Activities of Daily Living Scale; IMI, Intrinsic Motivation Inventory; M. Function Test, Manual Function Test; NHPT, Nine Hole Peg Test; PACES, Physical Activity Enjoyment Scale; ROM, Range of Motion; SIS, Stroke Impact Scale; SRMS, Stroke Rehabilitation Motivation Scale; SULCS, Stroke Upper Limb Capacity Scale; TUG, Timed up and Go Test; VAS, Visual Analog Scale; WMFT, Wolf Motor Function Test.

▴, favour intervention; ◄►, no effect; ▾, favour control.

1 Cross-over trial.

2 Median (Interquartile range).

3 Consensus formation.

Out of these 17 trials, 15 were RCTs (47–51, 54–63) and two were cross-over trials (52, 53). One cross-over trial with three arms was analysed as three separate comparisons, yielding 20 comparisons overall (53) (Table 1). 13 trials targeted upper extremity rehabilitation (47, 50–53, 56–63), two focused on lower extremity (48, 49), and two addressed trunk control (54, 55). The trials were conducted in 11 countries: Netherlands (*n* = 4), South Korea (*n* = 2), Taiwan (*n* = 2), USA (*n* = 2), Australia (*n* = 1), France (*n* = 1), Germany (*n* = 1), Spain (1), Malaysia (*n* = 1), Serbia (*n* = 1) and Thailand (*n* = 1). The total sample size comprised 521 stroke patients, with trial sizes ranging from 11 to 68 participants. Gender was reported in 15 trials, with 67% of participants being male. The mean age across trials was 60.3 years. Time post-stroke averaged 26.4 months (range 1.6 to 131.5 months), excluding one study that reported chronicity in rounded years only (57). Stroke severity ranged from mild to severe across trials, although inconsistent classification methods precluded direct summary and comparison. In 10 trials, both intervention and control were administered in clinical settings (47–49, 53, 54, 56, 57, 59, 60, 62), while five trials were conducted in home settings (50, 51, 55, 58, 63). One trial alternated delivery between clinic and home (52), and another trial had the intervention group train in the clinic, while the control group trained at home (61).

Among the interventions, 13 trials could be categorised as exergames—technology-based physical activity solutions (47, 49, 51–54, 56–62) (Supplementary Table S2). One trial included animal-assisted therapy (48), one smart technology with video supported exercises and reminder function (50), one music-supported therapy (63), and one classic boxing (55). Control group predominantly consisted of conditions that were similar to those of the intervention group, such as conventional physiotherapy or occupational therapy. In four trials, the content was identical to that of the intervention group (48, 49, 51, 60), differing only in one aspect, such as robot-assisted walking with or without a screen.

Intervention sessions duration ranged from 20 to 120 min. In one study, participants received a minimum of 20 min daily training with no specified maximum (51) and one study recommended training once to twice daily without further information about the duration time (50). Intervention frequency ranged from one to six days per week over two to 12 weeks. In 16 trials, dose and duration were equivalent across groups; only one trial allowed varied exercise time in controls (52).

Measurement instruments varied for motivation and motor performance (Table 1): Motivation was assessed using the IMI (*n* = 10) (25), Adherence (*n* = 5), PACES (*n* = 3) (27), self-developed questionnaires (*n* = 3), VAS for motivation (*n* = 2) and fun (*n* = 1), and the SRMS (*n* = 1) (26). Motor function was most commonly evaluated with the Box & Block Test (*n* = 5) (31), ARAT (*n* = 6) (30) and the WMFT (*n* = 3) (35), and motor impairment with the Fugl-Meyer-Test (*n* = 9) (28). Six trials reported the minimal clinically important difference (49, 51, 52, 55, 62, 63). Assessments of motor performance were conducted before and after the intervention. Regarding motivation, this approach was applied in four trials, whereas in all other trials the assessments were carried out after the intervention.

### Risk of bias

**Figure 2:**
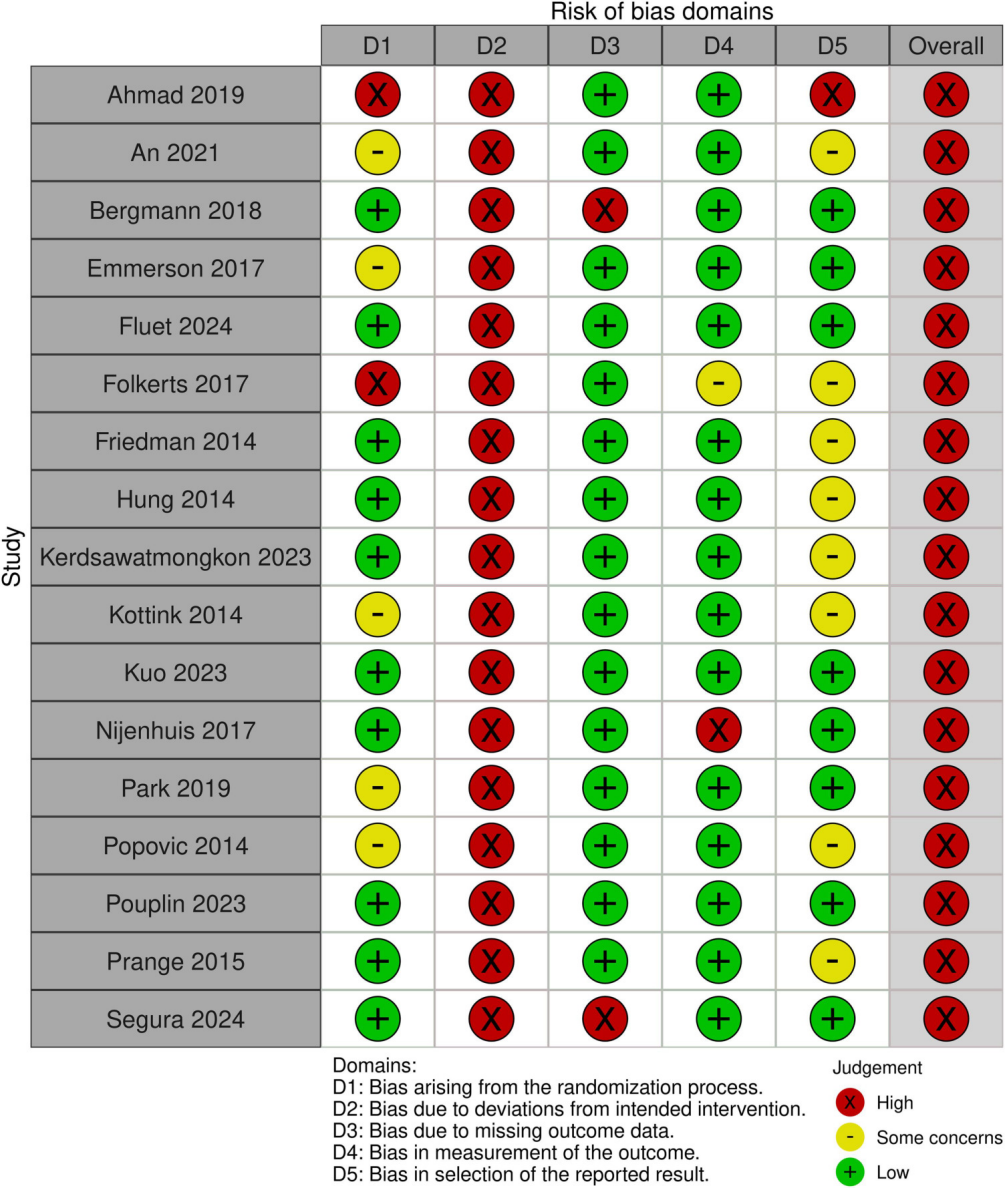
Risk of bias assessment.

The overall risk of bias was judged to be high across all included trials (Figure 2). Notably, bias related to allocation to intervention was inevitable, as participants blinding to the assigned intervention was generally not feasible. Except for this domain, the remaining four domains present a mixed picture. Only three studies (51, 57, 61) showed low risk across the other four domains, whereas half of the included studies raised concerns about selective reporting in domain four. Overall, this suggests low confidence in the evidence from these studies.

### Effect direction of motivation and motor function

The results of the comparative analyses are summarized in Table 1. In the domain of motivation, seven trials demonstrated a ‘favour intervention’ effect in at least one outcome (48, 49, 53, 54, 57, 59, 60), whereas one trial demonstrated a ‘favour control’ effect (58). In the domain of motor performance, six trials showed a ‘favour intervention’ effect in at least one outcome (48, 53, 57, 59, 60, 63). Five trials (48, 53, 57, 59, 60) favoured the intervention on both motivation and motor performance in at least one assessment. Among these, four trials involved exergames, while the remaining one involved animal-assisted therapy.

## Discussion

This systematic review identified 17 trials that assessed motor performance in adults post-stroke and simultaneously evaluated the effects on motivation (Table 1). Seven trials demonstrated a positive trend for motivation in at least one outcome, while six trials showed such a trend for motor performance. In total, five trials reported improvements in both motor performance and motivation in stroke patients in at least one outcome. These findings suggest that motor training interventions that enhance patients' motivation may contribute to improved rehabilitation outcomes.

One rational for this review was the notion that motivational benefits of training-based interventions are often implicitly assumed, but not necessarily tested. The systematic review of clinical trials that simultaneously assessed changes in motor performance and motivation substantiated this notion. Notably, more than half of the trials assessed in the full-text screening were excluded because they did not assess motivation as an outcome (Figure 1), despite claiming that the intervention would be motivating. A recent scoping review also indicates that exergames in gait rehabilitation are frequently linked to increased enjoyment by participants, but these effects are mostly assumed and not routinely measured or analyzed (17). Gamified devices have been identified in another review as having a gap in the consistent assessment of motivation (23). There is also a review that report motivational effects of VR-based systems solely based on measurements of quality of life, without any of the included trials employing a specific instrument designed to measure motivation directly (64). This underscores that motivation is currently still insufficiently measured in clinical trials, although there is the rationale that motivation plays an important role in rehabilitation.

A lack of available assessments cannot be the reason for the frequent discrepancy between claiming and measuring effects on motivation as highlighted above. Several instruments exist for assessing motivation (21). Since motivation comprises multiple constructs, various assessments focus on different aspects. The PACES measures the degree of pleasure or enjoyment a person experiences during or after physical activity. The SRMS is specifically designed for stroke patients and covers extrinsic and intrinsic motivation as well as motivation and amotivation (absence of motivation). The IMI is more commonly used in stroke rehabilitation (21), as was the case in this review; it is not specific to any patient group and focuses primarily on intrinsic factors. Adherence is also used as a marker of motivation. Therapy time is the most frequently used measure of therapy intensity (65), and higher motivation is closely associated with longer training durations (12, 66). Thus, adherence may be a consequence of motivation. It appears that a broad range of assessments is available for measuring different constructs of motivation. Clearly, a differential analysis of those assessments construct level would be desirable, but because of the low number of studies this is not sensible. This would, however, be an important aspect to investigate in future studies.

In this review, only scales directly measuring constructs of motivation, were included. During the screening process, it was observed that depression scales and quality of life questionnaires were frequently used. Although depression undoubtedly plays an role in the area of motivation, it was not considered as a construct in this review because it is a diagnosis of a pathological condition affecting motivation and mood (67). Inclusion of scales assessing depression, apathy, or quality of life would likely have resulted in a greater number of included trials.

Overall, the systematic review identified only a few interventions that may be classified to have measurable effects confer positive effects on both motivation and motor performance. Moreover, because of high risk of bias this small evidence base is also weak. The training content of the intervention groups and the control groups was largely comparable and differed mainly in the integration of motivational elements such as including a virtual reality component. It was assumed that patients in the intervention group would experience greater enjoyment during therapy and engage more intensively, which could have a positive impact on motor performance. This could only be observed in a small number of trials.

In neurorehabilitation, the enhancement or preservation of motor function is one of the major target parameters. A number of principles have been demonstrated to positively influence neuroplasticity and, consequently, motor function (68). Some of these principles can be implemented in exergames, as used by the majority of the included trials. Exergames are typically interactive video games set in an enriched environment that aim to promote physical activity. In the field of serious games, which are categorized as exergames, a meta-analysis demonstrated that they can significantly improve motor function as well as activity and participation in stroke patients (69). Principles that may foster plasticity in this context include repetitive and task-oriented practice, difficulty adjustment (shaping), the provision of variable training scenarios, and tracking-based feedback components that provide insights into performance and outcomes (68). It remains unclear to what extent each individual principle contributes to overall effectiveness, but the provision of feedback appears to be an important factor (69). Moreover, feedback is closely related to motivation (70, 71). Notably, all 13 exergame trials included in the systematic review incorporated feedback components, but only seven of them seemed to succeed in positively influencing motivation (Table 1). It is assumed that motivation can be enhanced through feedback when opportunities for successful experiences, perceived efficacy, autonomy, and competition are present (72). The study by Fluet and colleagues (51) examined two differentiated feedback conditions. In the ‘scaffolding’ condition, the difficulty level was increased when the player was successful. Players were explicitly shown the adaptive development of their skills (increasing or decreasing difficulty levels), whereas the control group received a more subtle, algorithm-based adaptation. It was expected that an appropriate challenge, combined with a developed sense of self-efficacy and clear knowledge of performance feedback, would lead to increased motivation. However, no significant differences were observed between groups regarding motor function, therapy adherence, or intrinsic motivation.

Another principle to regain motor function that is associated with neuroplasticity is social interaction (68). Social interaction plays also an important factor in the area of motivation (73). In line with this idea one of the five trials identified in this review that reported positive effects on motor function and motivation showed strong benefits of gait parameter and psychological variables when walking in the company of dogs (48). Animal-assisted therapy has been shown to positively influence both physical and mental health (74, 75), and animals may also function as social partners. Based on the trials identified in this review, the principle of social interaction has not yet been sufficiently considered in the development of exergames in neurorehabilitation. Thus, all included exergame trials were conducted in single-player modes without a competitive character or partner. A comparison between a multi-user virtual reality system and a single-user system using VR headsets, which was not included in this review, showed that participants spent significantly more time engaged in the activity and performed more arm movements when training together with others (represented by avatars) compared to training alone (76). Cognitive training trials conducted in competitive multi-user environments also demonstrated better outcomes in cognitive abilities and a higher enjoyment than single-user training (77). Another study identified in this review combined playing musical instruments in a home-based setting with a weekly videoconference with other participants (63). In addition, feedback and level adaptations were implemented via an app. At the impairment level, improvements were observed compared to the control group, which performed a motor arm training program. The IMI did not show overall superiority, but the Interest/Enjoyment subscale revealed a significant difference. These findings may be explained by the social aspects inherent in this particular intervention and hence strengthen the hypothesis that social aspects of rehabilitation training may be a critical modulator of motivation. Future developments of exergames and VR scenarios should therefore consider training with a multi-player mode to support motivational aspects to extend training sessions. Further trials about the principles are needed to more precisely investigate the specific mechanisms contributing to motivation enhancement and motor performance. Given the low evidence quality of the included studies, explicit statements about mechanisms of action cannot be made.

In future it would be desirable for trials to place greater emphasis on measuring motivation and to do so with explicit reference to their respective construct of motivation in reference to assumed treatment mechanisms. In the trials included in the review, only self-assessment measures were used. But the distinction between self-assessment and external assessment warrants greater consideration, particularly given evidence suggesting potential differences between self-reported and observer-based measures of motivation in stroke patients (12). Furthermore, multiple assessments should be employed to capture the various constructs of motivation. Moreover, trials need to examine longer intervention periods. The trials identified in this review lasted up to ten weeks, whereas rehabilitation in real life settings may extend over several months or years, requiring interventions that maintain motivation over longer periods.

### Limitations

Although this review was able to identify and discuss some potential mechanisms of motivation in stroke rehabilitation, it has several limitations. First, the data could not be pooled and analyzed in a meta-analysis due to the heterogeneity of outcome measures, particularly for motivation. Therefore, we decided to transfer the ratings into a direction of effect (44–46). On this basis, however, it was not taken into account which outcome measures represented the primary or most robust assessment. This could lead to an over-adjustment of the results.

In this study, purely psychologically oriented databases were not included, as motor function represents the primary endpoint alongside motivational aspects. Such databases typically do not index motor-focused studies, so only few additional relevant hits were to be expected. Consequently, the results primarily cover evidence from the perspective of motor neurorehabilitation point of view.

Further difficulties in comparability are reflected in the evaluation of assessments with subcategories, e.g., the IMI, where a varying number of subcategories could be selected. This means that some trials, for example, used four subcategories in the IMI, while others employed six subcategories. In this review, a majority voting principle was applied, i.e., if three out of five subcategories were significant, the entire assessment was classified as favouring the intervention. The results should therefore be interpreted only as indicative of a trend, since no weighting of subcategories was performed.

In the risk of bias assessment, all trials were rated negatively overall. This results from the fact that it is not possible to conduct motor rehabilitation interventions in a blinded fashion, as group allocation inevitably becomes obvious to participants at the latest by the start of the intervention. This could lead to bias, as participants might alter their behaviour if they were not assigned to the desired group, or the investigators might be influenced in their evaluations. The overall impression of the trials may therefore be somewhat distorted. This limitation, however, applies to virtual all trials in neurorehabilitation. In addition, the predominantly small sample sizes of the included trials further restrict the statistical power of the analyses.

## Conclusion

In summary, only a few motor rehabilitation interventions were identified that reported improvements in both patients' motivation and motor performance after stroke. It seems that exergames have potential; however, their underlying mechanisms of action remain insufficiently understood. Social interaction, which has proven effective in one study in animal-assisted therapy, could likewise be more widely integrated into the development of exergames for neurorehabilitation. More studies with higher levels of evidence are needed to enable more specific statements. Overall, research has not yet fully acknowledged the importance of motivation or the need for its standardized assessment.
