# Supplementary material for: Motivation in motor training interventions after stroke—a systematic review
Source: Front Rehabil Sci. 2026 May 28;7:1754746. doi: 10.3389/fresc.2026.1754746 (PMC13253544; doi:10.3389/fresc.2026.1754746)
Supplement: Supplementary file 1 [file Table1.pdf]

Supplemental Table S1: Searchstrategy Embase Ovid

Search	
1	motivat*.ti,ab.
2	exp motivation/
3	engage*.ti,ab.
4	"patient? adherence".ti,ab.
5	enjoy*.ti,ab.
6	"self-efficacy".ti,ab.
7	fun.ti,ab.
8	mood.ti,ab.
9	1 or 2 or 3 or 4 or 5 or 6 or 7 or 8
10	cerebrovascular disease/ or exp basal ganglion hemorrhage/ or exp brain ischemia/ or exp carotid artery diseases/ or exp cerebral artery disease/ or exp intracranial arteriovenous malformations/ or exp "intracranial embolism and thrombosis"/ or exp brain hemorrhages/ or exp stroke/ or exp brain infarction/
11	brain injuries/ or brain injury, chronic/
12	"cerebrovascular disorder".ti,ab.
13	exp cerebrovascular disease/
14	stroke.ti,ab.
15	poststroke.ti,ab.
16	post-stroke.ti,ab.
17	cerebrovasc$.ti,ab.
18	cerebral vascular.ti,ab.
19	((cerebral or cerebellar or brain$ or vertebrobasilar) adj5 (infarct$ or isch?emi$ or thrombo$ or emboli$ or apoplexy)).ti,ab.
20	((cerebral or brain or subarachnoid) adj5 (haemorrhage or hemorrhage or haematoma or hematoma or bleed$)).ti,ab.
21	10 or 11 or 12 or 13 or 15 or 16 or 17 or 18 or 19 or 20
22	"motor function*".ti,ab.
23	hemip*.ti,ab.
24	exp hemiplegia/ or exp paresis/
25	"functional abilit*".ti,ab.
26	"upper extremit*".ti,ab.
27	arm?.ti,ab.
28	"lower extremit*".ti,ab.
29	leg?.ti,ab.
30	gait.ti,ab.
31	walk*.ti,ab.
32	ambulation.ti,ab.
33	locomotion.ti,ab.
34	mobilit*.ti,ab.
35	balanc*.ti,ab.
36	stand*.ti,ab.
37	cycl*.ti,ab.
38	transfer*.ti,ab.
39	fall*.ti,ab.
40	"range of motion".ti,ab.
41	22 or 23 or 24 or 25 or 26 or 27 or 28 or 29 or 30 or 31 or 32 or 33 or 34 or 35 or 36 or 37 or 38 or 39 or 40
42	therap*.ti,ab.
43	treat*.ti,ab.
44	train*.ti,ab.
45	exp physiotherapy/
46	"occupational therap*".ti,ab.

47	exp occupational therapy/
48	physiotherap*.ti,ab.
49	rehabilitation.ti,ab.
50	exp rehabilitation/
51	42 or 43 or 44 or 45 or 46 or 47 or 48 or 49 or 50
52	9 and 21 and 41 and 51
53	exp randomized controlled trial/
54	controlled clinical trial/
55	random$.ti,ab.
56	randomization/
57	intermethod comparison/
58	placebo.ti,ab.
59	(compare or compared or comparison).ti,ab.
60	((evaluated or evaluate or evaluating or assessed or assess) and (compare or compared or comparing or comparison)).ab.
61	(open adj label).ti,ab.
62	((double or single or doubly or singly) adj (blind or blinded or blindly)).ti,ab.
63	double blind procedure/
64	parallel group$1.ti,ab.
65	(crossover or cross over).ti,ab.
66	((assign$ or match or matched or allocation) adj5 (alternate or group$1 or intervention$1 or patient$1 or subject$1 or participant$1)).ti,ab.
67	(assigned or allocated).ti,ab.
68	(controlled adj7 (study or design or trial)).ti,ab.
69	(volunteer or volunteers).ti,ab.
70	human experiment/
71	trial.ti.
72	"control group".ti,ab.
73	or/53-72
74	(random$ adj sampl$ adj7 ("cross section$" or questionnaire$1 or survey$ or database$1)).ti,ab. not (comparative study/ or controlled study/ or randomi?ed controlled.ti,ab. or randomly assigned.ti,ab.)
75	Cross-sectional study/ not (exp randomized controlled trial/ or controlled clinical study/ or controlled study/ or randomi?ed controlled.ti,ab. or control group$1.ti,ab.)
76	(((case adj control$) and random$) not randomi?ed controlled).ti,ab.
77	systematic review.ti,ab. not (trial or study).ti.
78	(nonrandom$ not random$).ti,ab.
79	"random field$".ti,ab.
80	(random cluster adj3 sampl$).ti,ab.
81	(review.ab. and review.pt.) not trial.ti.
82	"we searched".ab. and (review.ti. or review.pt.)
83	"update review".ab.
84	(databases adj4 searched).ab.
85	(rat or rats or mouse or mice or swine or porcine or murine or sheep or lambs or pigs or piglets or rabbit or rabbits or cat or cats or dog or dogs or cattle or bovine or monkey or monkeys or trout or marmoset$1).ti. and animal experiment/
86	animal experiment/ not (human experiment/ or human/)
87	or/74-86
88	73 not 87
89	52 and 88
