# Supplementary material for: Motivation in motor training interventions after stroke—a systematic review
Source: Front Rehabil Sci. 2026 May 28;7:1754746. doi: 10.3389/fresc.2026.1754746 (PMC13253544; doi:10.3389/fresc.2026.1754746)
Supplement: Supplementary file 2 [file Table2.docx]

Supplemental Table S2: Exergame characteristics

| **Study** | **Body Part** | **Device** | **Visualisation** | **Feedback** | **Level adaption** | **Immersivity** | **Product- type** |
| --- | --- | --- | --- | --- | --- | --- | --- |
| Ahmad 2019 | Upper  Limb | Custom made handle-bar incorporated into the Cy-Wee Z game controller | PC screen | yes:  auditory feedback | yes | semi- immersiv | custom |
| Bergmann  2018 | Lower  Limb | Lokomat® Hocoma | PC screen | yes: performance | yes | semi- immersiv | medical device |
| Fluet 2024 | Upper Limb | Home Virtual Rehabilitation System, Leap Motion controller | PC screen | yes:  explicit | yes | semi- immersiv | custom |
| Folkerts  2017 | Upper  Limb | Able X game controller + weight | PC screen | not mentioned | yes | semi- immersiv | medical device |
| Friedmann 2014 | Upper  Limb | Sensorized glove | PC + audible | yes:  visual + auditory | no | semi- immersiv | custom |
| Hung  2014 | Trunk control | Nintendo Wii fit | PC screen | yes | yes | semi- immersiv | comercial |
| Kottink  2014 | Upper  Limb | Horizontal screen, Webcam, motion capture software | Screen | yes | yes | semi- immersiv | custom |
| Kuo  2023 | Upper  Limb | PABLO Tyromotion | PC screen | yes | yes | semi- immersiv | medical device |
| Nijenhuis  2017 | Upper  Limb | SCRIPT (dynamic wrist and hand orthosis) + SaeboMAS (Saebo Mobile Arm Support) + Sensors | PC screen | yes: performance | yes | semi- immersiv | custom |
| Park  2019 | Upper  Limb | TPS System (Pressure Sensor) | PC screen | yes | n.a. | semi- immersiv | custom |
| Popovic  2014 | Upper  Limb | Drawing board + planar manipulandum | PC screen | yes:  visual | yes | semi- immersiv | custom |
| Pouplin  2023 | Upper  Limb | Custom made cylindrical tangible object + touch table | Screen | yes | yes | semi- immersiv | custom |
| Prange  2015 | Upper  Limb | Armeo® Boom Hocoma + webcam + reflectiv marker | PC screen | yes | yes | semi- immersiv | custom |
